# Whole Blood Gene Expression Profiling in Preclinical and Clinical Cattle Infected with Atypical Bovine Spongiform Encephalopathy

**DOI:** 10.1371/journal.pone.0153425

**Published:** 2016-04-13

**Authors:** Elena Xerxa, Maura Barbisin, Maria Novella Chieppa, Helena Krmac, Elena Vallino Costassa, Paolo Vatta, Marion Simmons, Maria Caramelli, Cristina Casalone, Cristiano Corona, Giuseppe Legname

**Affiliations:** 1 Scuola Internazionale Superiore di Studi Avanzati (SISSA), Functional and Structural Genomics sector, Trieste, Italy; 2 Istituto Zooprofilattico Sperimentale del Piemonte Liguria e Valle d'Aosta, Torino, Italy; 3 Pathology Department, Animal and Plant Health Agency (Weybridge), New Haw, Addlestone, United Kingdom; University of Verona, ITALY

## Abstract

Prion diseases, such as bovine spongiform encephalopathies (BSE), are transmissible neurodegenerative disorders affecting humans and a wide variety of mammals. Variant Creutzfeldt-Jakob disease (vCJD), a prion disease in humans, has been linked to exposure to BSE prions. This classical BSE (cBSE) is now rapidly disappearing as a result of appropriate measures to control animal feeding. Besides cBSE, two atypical forms (named H- and L-type BSE) have recently been described in Europe, Japan, and North America. Here we describe the first wide-spectrum microarray analysis in whole blood of atypical BSE-infected cattle. Transcriptome changes in infected animals were analyzed prior to and after the onset of clinical signs. The microarray analysis revealed gene expression changes in blood prior to the appearance of the clinical signs and during the progression of the disease. A set of 32 differentially expressed genes was found to be in common between clinical and preclinical stages and showed a very similar expression pattern in the two phases. A 22-gene signature showed an oscillating pattern of expression, being differentially expressed in the preclinical stage and then going back to control levels in the symptomatic phase. One gene, *SEL1L3*, was downregulated during the progression of the disease. Most of the studies performed up to date utilized various tissues, which are not suitable for a rapid analysis of infected animals and patients. Our findings suggest the intriguing possibility to take advantage of whole blood RNA transcriptional profiling for the preclinical identification of prion infection. Further, this study highlighted several pathways, such as immune response and metabolism that may play an important role in peripheral prion pathogenesis. Finally, the gene expression changes identified in the present study may be further investigated as a fingerprint for monitoring the progression of disease and for developing targeted therapeutic interventions.

## Introduction

Transmissible spongiform encephalopathies (TSEs), or prion diseases, are a group of fatal neurodegenerative disorders, which affect humans and a wide variety of animals. They include Creutzfeldt–Jakob disease (CJD), Gerstmann–Sträussler–Scheinker syndrome (GSS) and fatal familial insomnia (FFI) in humans [1], scrapie in goats and sheep [2], chronic wasting disease (CWD) in cervids [3] and bovine spongiform encephalopathy (BSE) in cattle [4]. The etiological agent of TSEs is an abnormally folded isoform (PrP^Sc^) of the cellular prion protein (PrP^C^), which accumulates in the nervous and lymphoreticular systems during the progression of the disease [5]. PrP^Sc^ accumulation, neuronal loss, spongiosis and astrogliosis are common hallmarks of prion diseases [6]. Despite the fact that the pathological features of these diseases are well characterized, the molecular mechanisms and the signaling pathways underlying TSEs are largely unknown.

The appearance of BSE in the United Kingdom (UK) in 1986 [7] led to an increased interest in these diseases, especially because of its epidemic nature in the UK. Foodborne transmission of BSE prions to humans was observed in the 1990s with the appearance of a new variant form of CJD (vCJD) [8]. It has been shown experimentally that BSE prions have strain characteristics identical to those of prion isolates from human cases of vCJD [9]. So far, 229 cases of vCJD have been reported around the world [10]. In recent years, two atypical forms of BSE have been identified in several European countries [11], Japan [12, 13], the United States [14] and Canada [15]. The two atypical BSE strains are denoted as H-type BSE and L-type BSE (also named bovine amyloidotic spongiform encephalopathy, BASE) [16, 17]. The “H” and “L” identify the higher and lower electrophoretic mobility of the unglycosylated protease resistant PrP^Sc^ fragment, respectively [18]. So far, both atypical subtypes have been identified only in cattle that were at least eight years old [19]. In view of that, it has been postulated that, unlike classical BSE (cBSE), cases of atypical BSE may have risen spontaneously, although transmission through feed or the environment cannot be ruled out. Indeed, histopathological as well as immunohistochemical analyses showed that atypical forms of BSE can be experimentally transmitted to mice [20–22] as well as to cattle. Moreover, they differ from cBSE and from each other in terms of clinical features [23–25] and biochemical properties [26–28]. Interestingly, some recent studies showed that H- and L-type BSE prions may acquire cBSE–like properties during propagation in animals expressing homologous bovine prion protein [29] or during inter-species transmission [17, 30], respectively. These findings support the view that the epidemic BSE agent could have originated from atypical cattle prions. While cBSE cases are now rapidly disappearing as a result of appropriate measures to control animal feeding, more insight into atypical BSE would be necessary in order to carry out risk assessment and to adopt appropriate control measures.

Given the infective nature of prions, the identification of specific molecular signatures may be helpful for the development of preclinical diagnostic tests in order to prevent horizontal transmission of the disease and potentially to develop targeted therapies in humans. High-throughput genomic techniques, such as DNA microarrays and RNA-seq, are the most frequently used methodologies for the identification of differentially expressed genes [31]. Gene expression approaches were first applied for studying scrapie [32, 33], while for BSE, and particularly for atypical BSE, they have appeared only recently in the literature [34–36]. Rodent models have been widely employed for large-scale studies of prion diseases [37, 38]; however, it is of the utmost importance to extend these studies to the ruminant species naturally affected by these diseases. In particular, most analyses in cattle have been performed using central nervous system (CNS) tissues from infected animals. Such studies are certainly of relevance but are not particularly suitable for diagnostic purposes. Also, the large majority of these genomic studies have been focused on the cBSE infection, while very few data are available about the involvement of peripheral tissues in atypical BSE infected cattle [36]. Peripheral blood is a readily accessible source of biological information on disease status and it is a suitable tissue for prospective rapid diagnostic tests in animals and patients. The objective of the present study was to identify molecular patterns in whole blood of atypical BSE-infected cattle in both clinical and preclinical stages of the disease. Transcriptional changes were analyzed using microarray technology and data were validated by Reverse Transcriptase quantitative PCR (RT-qPCR).

## Materials and Methods

All procedures involving animals were approved by the Home Office of the UK government according to the Animal (Scientific Procedures) Act 1986 and in conformity with the institutional guidelines of the Istituto Zooprofilattico Sperimentale del Piemonte Liguria e Valle d'Aosta, Turin, Italy (IZSPLV), that were in compliance with national (D.L. no. 116, G.U. suppl. 40, Feb. 18, 1992, Circular No.8, G.U., 14 July 1994) and international regulations (EEC Council Directive 86/609, OJ L 358, 1 Dec.12, 1987). All the experimental protocols proposed were reviewed and approved by the IZSPLV Animal Care and Use Committee (IACUC).

### Blood Samples

Blood samples from 8 BSE-infected cattle (4 with H-type and 4 with L-type BSE) and 2 non-infected controls were provided by the Biological Archive Group at the Animal and Plant Health Agency, United Kingdom. All procedures involving animals were approved by the Home Office of the UK government according to the Animal (Scientific Procedures) Act 1986. The calves were born by crossing *Aberdeen angus* with females imported from Denmark (Danish Holstein, Danish milking red). The inoculation details have been reported previously [23]. Briefly, experimental cattle were intracerebrally inoculated with 1 ml of a 10% brain homogenate of either L-type or H-type BSE at 10–11 months of age [23]. All infected cattle used in this study were females. The negative controls were age and sex-matched with the infected group. For each animal, the blood sampling was performed at 2 different time points after inoculation, corresponding to the preclinical (6-months post infection) and the clinical (from 22 to 26 months post infection) stage of the disease. In this way we obtained 16 samples, 8 in the preclinical and 8 in the clinical stage. The estimated clinical onset after infection was based on the presence of changes in behavior, unexpected startle responses, and difficulty in rising [23]. Neurological examination and behavioral observations were conducted routinely until the culling of the animals. TSE infection was confirmed by post-mortem immunohistochemistry on brain sections of the animals [23]. Detailed information on the husbandry procedures and the pathological signs have been described in a previous study published by Konold et al. [23]. Finally, blood samples from 6 sex-matched *Aberdeen angus* from a different herd were added to the study and used as additional negative controls to obtain a sample size comparable to the one of the infected animals (8 samples).

### RNA Isolation

500 μL of fresh blood were stabilized in 1.3 mL RNA*later*^®^ Solution and immediately frozen at -20°C. Samples were sent in dry ice to IZSTO (Turin, Italy), where the RNA was isolated according to the RiboPure^™^-Blood Kit manufacturer’s instructions (Ambion^®^). DNase I treatment (Ambion^®^) was included in the RNA extraction protocol to reduce DNA contamination. Purified RNA was eluted in 50 μL elution solution and the final concentration, as well as the absence of protein, was determined using a Thermo Scientific^™^ NanoDrop 2000 spectrophotometer. Since the RNA concentration was too low to proceed with the subsequent analysis, the RNA samples were concentrated using a Labconco CentriVap concentrator. The new concentration was assessed using a Thermo Scientific^™^ NanoDrop 2000 spectrophotometer and the integrity of the RNA was determined by capillary electrophoresis (Agilent 2100 Bioanalyzer, Agilent Technologies, Santa Clara, USA).

### Microarray Hybridization, Statistical Analysis and Data Mining

24 RNA samples were used for the microarray analysis: 8 preclinical (P1, P2, P4, P5, P7, P8, EP9 and P10), 8 clinical (S1, S2, S3, S4, S7, S8, S9 and S10), and 8 control (c.2, c.3, c.P3, c.5, c.S5, C.P6, C.S6 and c.9) samples. 120 ng of each total RNA were used as template for the synthesis of biotin-labeled cRNA according to the standard one-cycle amplification and labeling protocol developed by Affymetrix (Santa Clara, CA). cRNA was then fragmented and hybridized on GeneChip^®^ Bovine Genome Array, which contains over 24128 probe sets. The microarrays were washed, stained (Affymetrix fluidic station 450 DX) and scanned (Affymetrix scanner 3000 7G). Cell intensity values from the raw array data were computed using the Affymetrix GeneChip^®^ Operating Software (GCOS). Microarray quality control and statistical analysis were performed in the software system *R* using the Bioconductor package OneChannelGUI [39–41]. The LIMMA algorithm was used to compute a linear model fit [42]. Data filtering and normalization was carried out using GC-Robust multi-array analysis (GCRMA) from imported Affymetrix data (.CEL) files. After the assessment and inspection of microarray quality controls (RNA degradation plot, RLE and NUSE plots) we identified one low quality control sample (cS5) and excluded it from the analysis (S1 Appendix). Gene probes with a *p* value ≤0.05 and fold-change ≥2 were considered to be differentially expressed. Differentially expressed probe sets were functionally classified using David Bioinformatics tool [43, 44] on the Affymetrix bovine background. Heat maps were generated using the heatmap.2 function from the gplots library in the *R* statistical environment [45, 46]. Probe set data were hierarchically clustered with complete linkage using the Euclidean metric.

### RT-qPCR

To confirm the microarray results, we performed RT-qPCR using SYBR^®^ green assay (Bio-Rad Laboratories, Inc.) for a selected number of target genes. The RT-qPCR analysis was performed on 22 samples (from 7 control, 8 preclinical and 7 clinical animals). For each sample, 250 ng of total RNA were used as template for the cDNA synthesis with the SuperScript^®^ III Reverse Transcriptase and the Oligo (dT)_20_ Primer (Life Technologies). PCR primers were designed using the online tool Primer-BLAST [47] provided by NCBI. Whenever possible, the primer pairs were designed in order to span an exon-exon junction, thus preventing the amplification of genomic DNA. The primer sequences were as follows: for GNLY 5’-AGC CCG ATG AGA ATA CCG TT and 5’- CGA TGT CCT CAG CGA TGG TA; for CD40L 5’-ACA ACC TCT GTT CTC CAG TG and 5’-GCT GTT TCC CGT TTT CGA GG; for PDK4 5’-TGG TGT TCC CCT GAG AGT CA and 5’-GTA ACC AAA ACC AGC CAG CG; for HBA2 5’- ACA AGG GCA ATG TCA AGG CC and 5’- TCG AAG TGG GGG AAG TAG GT; for XIST 5’-GTG GCA AGG ACC AGA ATG GA and 5’-TCC GAC CCC AGT ATT AGC CA; for GNB4 5’-AGA TCG TGC AGG TGT TCT TG and 5’-CTG TCC CAA GAC CCT GTT G; for IDO1 5’-ATT GGT GGA GTC CCT GCA GAC and 5’-CTG CAG GGT AGC ATT GCC TT; for BOLA 5’CTC GTA GTA GCT GTG GTG GC and 5’ACT GTC ACT GCT TGC AGC C; for SEL1L3 5’-TGA AGG AGT GGTTTC GCC TG and 5’ TTC AAA TCC TGC CCA GTG CT; for RPL12 5’-AGG GTC TGA GGA TTA CAG TGA AA and 5’-GAT CAG GGC AGA AGC AGA AGG; for ACTB 5’-GGA CTT CGA GCA GGA GAT GG and 5’-TTC CAT GCC CAG GAA GGA AG; for GAPDH 5’-AGG TCG GAG TGA ACG GAT TC and 5’-ATG GCG ACG ATG TCC ACT TT. The RT-qPCR reactions were carried out by denaturing at 95°C for 15s, annealing at 60°C for 1 min and extension at 55°C for 1 min for 45 cycles. Melt curve analysis and gel electrophoresis of amplification products were performed for each primer pair to confirm the production of a single PCR amplicon. The amplification was performed using a CFX96^™^ Real-Time PCR Detection System (Bio-Rad Laboratories, Inc.). All the RT-qPCR reactions were run in triplicate and included the following controls: no template (NTC) and minus-reverse transcriptase (RT-) negative controls. The normalization accuracy was improved by geometric averaging of multiple reference genes [48] and using two inter-run calibrators to reduce inter-run variation. We decided to use a normalization factor based on three reference genes (*GAPDH*, *RPL12* and *ACTB*) since it has been shown in the literature that this is the minimal number required for a reliable normalization [49]. Stability of the selected reference genes was determined by calculating their geNorm M value (M) and the coefficient of variation (CV) on the normalized relative quantities [50]. M and CV values were then compared against empirically determined thresholds for acceptable stability (~ 1 and ~ 0.5 for M and CV values respectively) [50] (S2 Appendix). The statistical analysis and the fold change calculation were carried out using qBasePlus 1.1 software [50].

## Results

### Identification of Differentially Expressed Genes (DEGs) in the Blood of Atypical BSE-Infected Cattle

To investigate if gene expression alterations were present in blood from atypical BSE-infected cattle (clinical and preclinical), we performed microarray experiments using Affymetrix GeneChip^®^ Bovine Genome Array. Since the goal of this project was to identify a common pattern of DEGs in atypical BSE infection, we defined the 4 H- and the 4 L- type inoculated-cattle as one single group of 8 animals named as atypical infected cattle. This approach allowed us to increase the sample size to improve the statistics and thus obtain more reliable results. The distribution of signal intensities, relative log expression (RLE) and normalized unscaled standard error (NUSE) plots were examined in order to avoid procedural failures and the presence of degraded RNA samples. After the assessment of microarray quality controls we identified one control sample (cS5) as an outlier and excluded it from the analysis (see S1 Appendix). Statistical analysis was performed on microarray results using the oneChannelGUI Bioconductor package [39]. The raw microarray data were deposited in the Gene Expression Omnibus (GEO) repository and assigned the accession number GSE69048. The data sets supporting the results of this article are available in the Gene Expression Omnibus (GEO) repository: http://www.ncbi.nlm.nih.gov/geo/query/acc.cgi?token=mzgxuaagddwftcz&acc=GSE69048.

Statistical comparison between the infected animals (clinical and preclinical) and the control group (IvsCtrl) revealed a total of 101 differentially regulated probe sets (*p* value lower than 0.05 and changes in expression higher than 2-fold) as shown in Table 1. Some of these probe sets encoded for the same gene. Gene annotation, performed using DAVID Bioinformatics Resources [43, 44], identified a subset of 93 genes with known functions. The most relevant functional groups are reported in Table 2.

**Table 1:** Differentially expressed genes found in infected animals versus control group by the microarray analysis^a^.

Probe ID	Gene symbol	Gene name	*P* value	FC
Bt.6653.2.A1_at	ALKBH4	alkB, alkylation repair homolog 4 (E. coli)	1.35E-07	-2.31687
Bt.8586.1.S1_at	LOC512150	myeloid-associated differentiation marker-like	4.17E-05	4.192159
Bt.21996.1.S1_at	IGHE	Immunoglobulin heavy constant epsilon	4.77E-05	-5.1041
Bt.16101.1.S1_s_at	GNLY /// LOC100300483	granulysin /// antimicrobial peptide NK-lysin-like	5.7E-05	-4.17735
Bt.28383.1.S1_at	GNLY	granulysin	8.98E-05	-4.83354
Bt.9265.2.S1_at	BATF	basic leucine zipper transcription factor, ATF-like	0.000112	-2.57096
Bt.14153.1.S1_at	NEB	nebulin	0.000145	-4.89569
Bt.16101.1.S1_at	GNLY	granulysin	0.000212	-4.81011
Bt.12986.1.S1_at	MAD2L1	MAD2 mitotic arrest deficient-like 1 (yeast)	0.000235	-2.72219
Bt.9265.1.A1_at	BATF	basic leucine zipper transcription factor, ATF-like	0.000236	-2.55269
Bt.23123.1.S1_at	BHLHE40	basic helix-loop-helix family, member e40	0.000575	-2.36861
Bt.26326.1.A1_at	MTBP	Mdm2, transformed 3T3 cell double minute 2, p53 binding protein (mouse) binding protein, 104kDa	0.000623	2.784763
Bt.22526.1.S1_at	HSPB8	heat shock 22kDa protein 8	0.000858	-2.39332
Bt.16916.3.S1_at	KLF11	Kruppel-like factor 11	0.000929	4.455542
Bt.24923.2.S1_a_at	SEL1L3	sel-1 suppressor of lin-12-like 3 (C. elegans)	0.001057	-3.34168
Bt.8804.1.S1_at	NELL2	NEL-like 2 (chicken)	0.00117	-2.34981
Bt.28654.1.S1_at	LOC100850906 /// USP42	ubiquitin carboxyl-terminal hydrolase 42-like /// ubiquitin specific peptidase 42	0.00132	3.549622
Bt.18321.1.A1_at	GNB4	guanine nucleotide binding protein (G protein), beta polypeptide 4	0.001338	8.267761
Bt.24630.2.S1_at	41527	septin 10	0.001368	-3.33002
Bt.21975.1.S1_at	PRF1	perforin 1 (pore forming protein)	0.001549	-2.00589
Bt.20330.1.S1_at	PRSS23	protease, serine, 23	0.001702	-3.42322
Bt.24929.1.A1_at	RG9MTD3	RNA (guanine-9-) methyltransferase domain containing 3	0.001896	2.420786
Bt.6968.1.S1_at	IPCEF1	Interaction protein for cytohesin exchange factors 1	0.001963	2.424841
Bt.9675.1.S1_at	LOC100847724	extracellular peptidase inhibitor-like	0.002117	-3.52792
Bt.21979.1.S1_at	CXCR6	chemokine (C-X-C motif) receptor 6	0.002164	-2.20884
Bt.9504.1.A1_at	CCL4	chemokine (C-C motif) ligand 4	0.002853	-2.65024
Bt.28040.1.S1_at	LOC781494	myeloid-associated differentiation marker-like	0.002999	-3.59576
Bt.23505.1.S1_at	PDK4	pyruvate dehydrogenase kinase, isozyme 4	0.003176	3.186119
Bt.24236.1.S1_at	DLC1	deleted in liver cancer 1	0.003559	2.297004
Bt.26636.1.S1_at	NKG7	natural killer cell group 7 sequence	0.003806	-2.16912
Bt.6147.1.S1_a_at	METTL12	methyltransferase like 12	0.00446	-2.15129
Bt.49.1.S1_at	CD40LG	CD40 ligand	0.004517	-3.23666
Bt.26259.1.A1_at	ZNF462	zinc finger protein 462	0.004806	3.969328
Bt.15915.1.S1_at	LRRC70	leucine rich repeat containing 70	0.005099	2.154164
Bt.17280.1.S1_at	PLEKHH1	pleckstrin homology domain-containing family H member 1-like	0.005117	2.356958
Bt.29009.1.A1_at	RYR3	ryanodine receptor 3	0.00525	-5.46377
Bt.2129.1.S1_at	LOC100850064	versican core protein-like	0.005936	2.001974
Bt.22415.2.A1_at	LOC512863	sialic acid-binding Ig-like lectin 14-like	0.006306	2.230891
Bt.19308.1.S1_at	BACH2	BTB and CNC homology 1, basic leucine zipper transcription factor 2	0.006597	3.175266
Bt.16861.1.A1_at	LOC515128	major facilitator superfamily domain-containing protein 4-like	0.006682	2.319431
Bt.22301.1.S1_at	ATP6V0A4	ATPase, H+ transporting, lysosomal V0 subunit a4	0.006816	-2.30662
Bt.15705.1.S1_at	DSTN	destrin (actin depolymerizing factor)	0.006907	2.216088
Bt.28637.1.S1_at	LOC100848843	myeloid-associated differentiation marker-like	0.007707	2.740488
Bt.24983.1.A1_at	DYNC2H1	dynein, cytoplasmic 2, heavy chain 1	0.008144	-2.08508
Bt.24543.1.A1_at	KCTD1	potassium channel tetramerisation domain containing 1	0.01017	2.673429
Bt.16916.1.S1_at	KLF11	Kruppel-like factor 11	0.010186	2.110118
Bt.11259.1.S1_at	IFI27	putative ISG12(a) protein	0.010909	3.109152
Bt.17081.2.S1_at	LMO2	LIM domain only 2 (rhombotin-like 1)	0.011572	-2.37451
Bt.22214.1.S1_at	CD180	CD180 molecule	0.011606	-2.04421
Bt.7145.1.S1_at	GZMB /// LOC100125946	granzyme B (granzyme 2, cytotoxic T-lymphocyte-associated serine esterase 1) /// uncharacterized LOC100125946	0.015009	-3.15552
Bt.20540.1.S1_at	CD79B	CD79b molecule, immunoglobulin-associated beta	0.015089	-3.58646
Bt.20257.2.S1_at	BCNT2	Bucentaur-2	0.015853	-2.28942
Bt.155.1.S1_at	IL8	interleukin 8	0.016	2.249822
Bt.24112.1.A1_at	CXHXorf57	chromosome X open reading frame, human CXorf57	0.016027	-2.76004
Bt.18253.1.A1_at	KIAA1324L	KIAA1324-like ortholog	0.016067	2.359824
Bt.27043.2.S1_at	FCER1A	Fc fragment of IgE, high affinity I, receptor for; alpha polypeptide	0.016226	-2.06287
Bt.13469.1.S1_at	SDSL	serine dehydratase-like	0.016296	-2.5064
Bt.9262.1.A1_at	SPIB	Spi-B transcription factor (Spi-1/PU.1 related)	0.016742	-3.49334
Bt.9974.1.S1_at	CCL3	chemokine (C-C motif) ligand 3	0.01705	-2.10999
Bt.19014.1.A1_at	NDUFS3	NADH dehydrogenase (ubiquinone) Fe-S protein 3, 30kDa (NADH-coenzyme Q reductase)	0.017446	4.152097
Bt.2899.1.S2_at	FOS	FBJ murine osteosarcoma viral oncogene homolog	0.017668	2.486687
Bt.6438.1.A1_at	TGFB2	transforming growth factor, beta 2	0.019127	3.436742
Bt.3352.1.S1_at	ASIP	agouti signaling protein	0.019681	-2.09284
Bt.9163.2.S1_at	P2RY10	purinergic receptor P2Y, G-protein coupled, 10	0.019736	-2.0468
Bt.27760.1.S1_at	BoLA /// BOLA-A	major histocompatibility complex, class I, A /// major histocompatibility complex, class I, A	0.02069	-3.40943
Bt.3805.1.S1_at	BOLA-N /// JSP.1 /// LOC100125916	MHC class I antigen /// MHC Class I JSP.1 /// uncharacterized protein 100125016	0.021303	3.897296
Bt.17019.1.A1_at	FOXP1	Forkhead box P1	0.021388	2.003436
Bt.20640.1.A1_at	C22H3orf64	chromosome 22 open reading frame, human C3orf64	0.021803	2.039162
Bt.411.1.S1_at	NRG1	neuregulin 1	0.021883	2.757968
Bt.11847.1.A1_at	XIST	X (inactive)-specific transcript	0.023013	-21.2975
Bt.24923.1.S1_at	SEL1L3	sel-1 suppressor of lin-12-like 3 (C. elegans)	0.023619	-3.25125
Bt.13367.1.A1_at	XIST	X (inactive)-specific transcript	0.024218	-34.6725
Bt.29820.1.S1_s_at	BOLA	MHC class I heavy chain	0.024899	-6.08329
Bt.23911.1.A1_at	XIST	X (inactive)-specific transcript	0.025193	-29.733
Bt.22139.1.S1_at	COBLL1	COBL-like 1	0.026423	-2.76573
Bt.22854.1.S1_at	CA2	carbonic anhydrase II	0.026826	-2.33612
Bt.13330.1.S1_at	PDK4	pyruvate dehydrogenase kinase, isozyme 4	0.02815	2.399512
Bt.16070.2.S1_at	LOC786352	apolipoprotein L, 1-like	0.028855	-2.31442
Bt.17473.2.S1_at	RPE	ribulose-5-phosphate-3-epimerase	0.030357	2.209343
Bt.16966.1.S1_at	CXCL10	chemokine (C-X-C motif) ligand 10	0.030459	-3.06647
Bt.23172.1.S1_at	BAX	BCL2-associated X protein	0.030653	-2.28799
Bt.4609.1.S1_at	LOC100847474	uncharacterized LOC100847474	0.031831	-2.619
Bt.20687.1.A1_at	C5H12orf35	chromosome 5 open reading frame, human C12orf35	0.032384	2.091363
Bt.3651.2.A1_at	C15H11orf96	chromosome 15 open reading frame, human C11orf96	0.035164	2.092887
Bt.5408.1.A1_at	UCHL1	ubiquitin carboxyl-terminal esterase L1 (ubiquitin thiolesterase)	0.035211	2.097092
Bt.7015.1.S1_at	EED	embryonic ectoderm development	0.036537	-2.08921
Bt.14010.1.S1_at	PTGR1	prostaglandin reductase 1	0.03669	-2.19439
Bt.2899.1.S1_at	FOS	FBJ murine osteosarcoma viral oncogene homolog	0.03784	2.465631
Bt.1075.1.A1_at	ZNF24	zinc finger protein 24	0.038258	2.707935
Bt.20494.1.S1_at	RYBP	RING1 and YY1 binding protein	0.038552	2.157803
Bt.27261.2.S1_at	LOC100847574	multidrug resistance-associated protein 4-like	0.040322	3.296289
Bt.23094.1.A1_at	AKR1C4 /// LOC506594	aldo-keto reductase family 1, member C4 (chlordecone reductase; 3-alpha hydroxysteroid dehydrogenase, type I; dihydrodiol dehydrogenase 4) /// prostaglandin F synthase 1-like	0.041102	-2.13672
Bt.20540.3.A1_at	CD79B	CD79b molecule, immunoglobulin-associated beta	0.04229	-2.06338
Bt.24372.1.S1_at	ZRSR2Y	zinc finger (CCCH type), RNA-binding motif and serine/arginine rich 2	0.042712	3.786694
Bt.7122.1.S1_at	HELZ	helicase with zinc finger	0.043553	2.564275
Bt.213.1.S1_at	CD163L1	CD163 molecule-like 1	0.047294	2.499982
Bt.20216.2.S1_at	RPIA	ribose 5-phosphate isomerase A	0.048256	-2.06759
Bt.27759.2.S1_at	IDO1	indoleamine 2,3-dioxygenase 1	0.048525	-3.72942
Bt.29815.1.S1_x_at	BOLA	MHC class I heavy chain	0.048538	-3.12234
Bt.15705.1.S2_at	DSTN	destrin (actin depolymerizing factor)	0.048589	2.055416
Bt.16933.2.A1_at	LOC100298891	aTP-binding cassette, sub-family C (CFTR/MRP), member 4-like	0.049066	2.747912

^a^FC = fold change.

**Table 2:** Functional classification of differentially expressed genes in blood of infected cattle versus control group^a^.

Pathway	Probe name	Gene symbol	Gene name	*P* value	Fold Enrichment
**Autoimmune thyroid disease**	BT.21975.1.S1_AT	PRF1	perforin 1 (pore forming protein)	1.01E-04	19.14728682
	BT.29815.1.S1_X_AT	BOLA	MHC class I heavy chain		
	BT.29820.1.S1_S_AT	BOLA	MHC class I heavy chain		
	BT.3805.1.S1_AT	BOLA-N /// JSP.1 /// LOC100125916	MHC class I antigen /// MHC Class I JSP.1 /// uncharacterized protein 100125016		
	BT.49.1.S1_AT	CD40LG	CD40 ligand		
	BT.7145.1.S1_AT	GZMB /// LOC100125946	granzyme B (granzyme 2, cytotoxic T-lymphocyte-associated serine esterase 1) /// uncharacterized LOC100125946		
**Extracellular region**	BT.155.1.S1_AT	IL8	interleukin 8	2.22E-04	3.036995516
	BT.16070.2.S1_AT	LOC786352	apolipoprotein L, 1-like		
	BT.16966.1.S1_AT	CXCL10	chemokine (C-X-C motif) ligand 10		
	BT.20330.1.S1_AT	PRSS23	protease, serine, 23		
	BT.20640.1.A1_AT	C22H3orf64	chromosome 22 open reading frame, human C3orf64		
	BT.2129.1.S1_AT	LOC100850064	versican core protein-like		
	BT.213.1.S1_AT	CD163L1	CD163 molecule-like 1		
	BT.29009.1.A1_AT	RYR3	ryanodine receptor 3		
	BT.49.1.S1_AT	CD40LG	CD40 ligand		
	BT.6438.1.A1_AT	TGFB2	transforming growth factor, beta 2		
	BT.8804.1.S1_AT	NELL2	NEL-like 2 (chicken)		
	BT.9504.1.A1_AT	CCL4	chemokine (C-C motif) ligand 4		
	BT.9974.1.S1_AT	CCL3	chemokine (C-C motif) ligand 3		
	BT.3352.1.S1_AT	ASIP	agouti signaling protein		
**Immune response**	BT.155.1.S1_AT	IL8	interleukin 8	4.91E-04	5.451442755
	BT.16966.1.S1_AT	CXCL10	chemokine (C-X-C motif) ligand 10		
	BT.27760.1.S1_AT	BoLA /// BOLA-A	major histocompatibility complex, class I, A /// major histocompatibility complex, class I, A		
	BT.29815.1.S1_X_AT	BOLA	MHC class I heavy chain		
	BT.29820.1.S1_S_AT	BOLA	MHC class I heavy chain		
	BT.3805.1.S1_AT	BOLA-N /// JSP.1 /// LOC100125916	MHC class I antigen /// MHC Class I JSP.1 /// uncharacterized protein 100125016		
	BT.49.1.S1_AT	CD40LG	CD40 ligand		
	BT.9504.1.A1_AT	CCL4	chemokine (C-C motif) ligand 4		
	BT.9974.1.S1_AT	CCL3	chemokine (C-C motif) ligand 3		
**Chemokine activity**	BT.155.1.S1_AT	IL8	interleukin 8	5.42E-04	24.18039216
	BT.16966.1.S1_AT	CXCL10	chemokine (C-X-C motif) ligand 10		
	BT.9504.1.A1_AT	CCL4	chemokine (C-C motif) ligand 4		
	BT.9974.1.S1_AT	CCL3	chemokine (C-C motif) ligand 3		
**Locomotory behavior**	BT.155.1.S1_AT	IL8	interleukin 8	0.001387806	9.982954545
	BT.16966.1.S1_AT	CXCL10	chemokine (C-X-C motif) ligand 10		
	BT.5408.1.A1_AT	UCHL1	ubiquitin carboxyl-terminal esterase L1 (ubiquitin thiolesterase)		
	BT.9504.1.A1_AT	CCL4	chemokine (C-C motif) ligand 4		
	BT.9974.1.S1_AT	CCL3	chemokine (C-C motif) ligand 3		
**Inflammatory response**	BT.155.1.S1_AT	IL8	interleukin 8	0.001604508	9.598994755
	BT.16966.1.S1_AT	CXCL10	chemokine (C-X-C motif) ligand 10		
	BT.49.1.S1_AT	UCHL1	ubiquitin carboxyl-terminal esterase L1 (ubiquitin thiolesterase)		
	BT.9504.1.A1_AT	CCL4	chemokine (C-C motif) ligand 4		
	BT.9974.1.S1_AT	CCL3	chemokine (C-C motif) ligand 3		
**Antigen processing and presentation via MHC class I**	BT.27760.1.S1_AT	BoLA /// BOLA-A	major histocompatibility complex, class I, A /// major histocompatibility complex, class I, A	0.002359977	39.93181818
	BT.29820.1.S1_S_AT	BOLA	MHC class I heavy chain		
	BT.3805.1.S1_AT	BOLA-N /// JSP.1 /// LOC100125916	MHC class I antigen /// MHC Class I JSP.1 /// uncharacterized protein 100125016		
	BT.29815.1.S1_X_AT	BOLA	MHC class I heavy chain		
**B cell proliferation**	BT.23172.1.S1_AT	BAX	BCL2-associated X protein	0.0337752778987117	57.0454545454545
	BT.49.1.S1_AT	CD40LG	CD40 ligand		
**Cell adhesion molecules (CAMs)**	BT.2129.1.S1_AT	LOC100850064	versican core protein-like	0.038771205	5.105943152
	BT.29820.1.S1_S_AT	BOLA	MHC class I heavy chain		
	BT.3805.1.S1_AT	BOLA-N /// JSP.1 /// LOC100125916	MHC class I antigen /// MHC Class I JSP.1 /// uncharacterized protein 100125016		
	BT.49.1.S1_AT	CD40LG	CD40 ligand		
	BT.29815.1.S1_X_AT	BOLA	MHC class I heavy chain		
**Regulation of secretion**	BT.27043.2.S1_AT	FCER1A	Fc fragment of IgE, high affinity I, receptor for; alpha polypeptide	0.048207557	8.319128788
	BT.49.1.S1_AT	CD40LG	CD40 ligand		
	BT.29009.1.A1_AT	RYR3	ryanodine receptor 3		

^a^ The gene enrichment analysis was performed using DAVID bioinformatics tool 6.7 (NIAID/NIH, USA). Only genes with a known GO and belonging to the most relevant functional categories are represented in the list.

**Fig 1:**
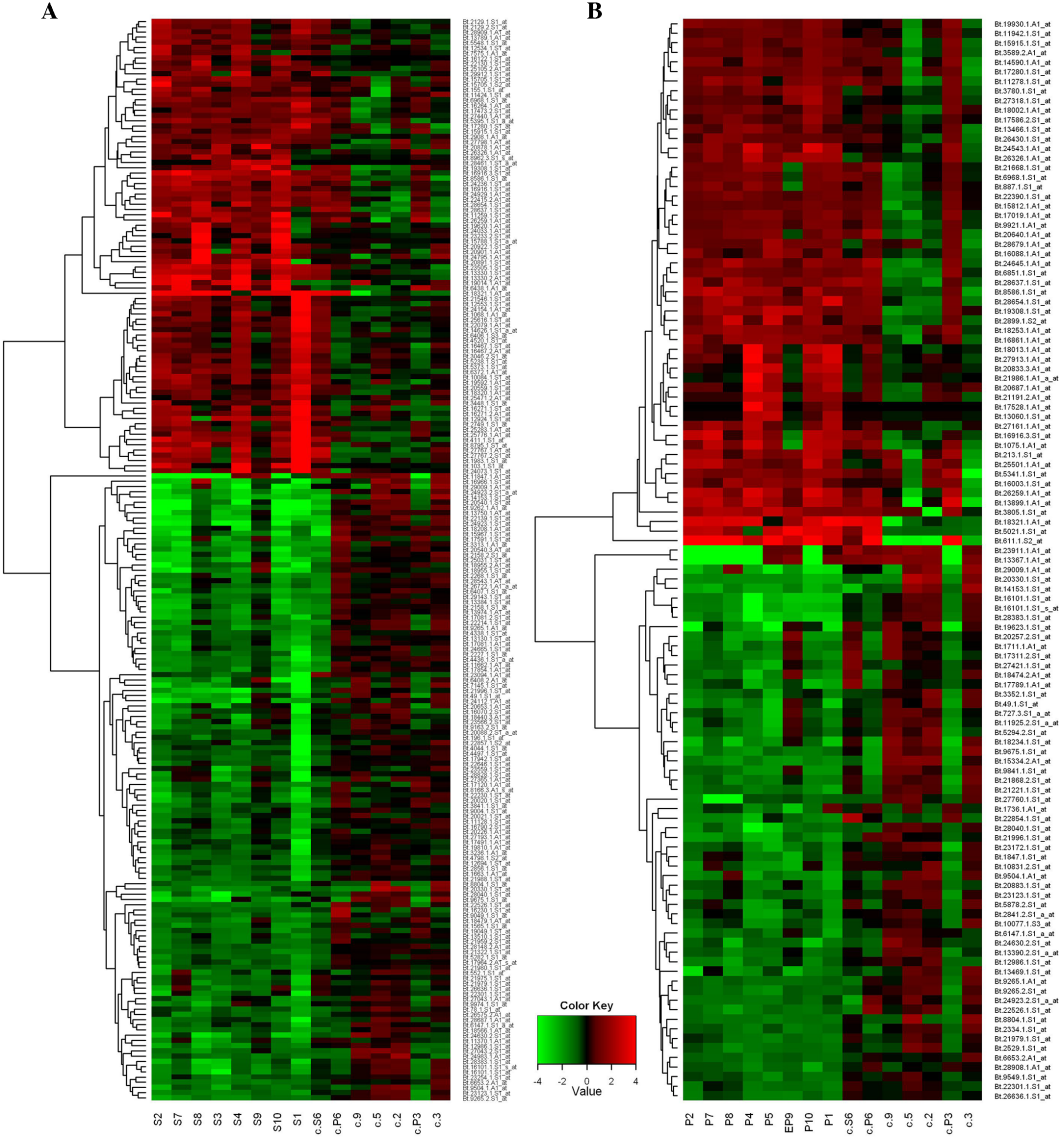
Heat maps representing the DEGs found in clinical and preclinical cattle with atypical BSE. Two heat maps were generated using the *heatmap*.*2* function from the *gplots* library in *R* statistical environment. DEGs were hierarchically clustered with complete linkage using the Euclidean metric. The heat maps represent the most significant DEGs (p value ≤.0.05 and fold change ≥ 2) in clinical (A) and preclinical (B) animals compared to the control group. Animals are reported in the x-axis while the differentially expressed probes are in the y-axis.

To evaluate to what extent gene expression alterations in blood were related to the preclinical or clinical stage of the disease, two distinct statistical analyses were performed comparing each group (8 samples) with the control one: clinical versus control (CvsCtrl) and preclinical versus control (PvsCtrl). In the clinical stage, a total of 207 probe sets showed significant alteration in expression levels compared to the control group. Among these, 87 were up-regulated while 120 had a reduction in expression. Interestingly, a pronounced alteration in the gene expression profile was also found in the preclinical stage, with a total number of 113 differentially expressed probe sets (55 genes were up-regulated while 58 were down-regulated). Two heat maps representing the differentially expressed probe sets in preclinical and clinical groups are shown in Fig 1. The complete probe set lists with the relative p values and fold changes can be found in S1 and S2 Tables.

**Fig 2:**
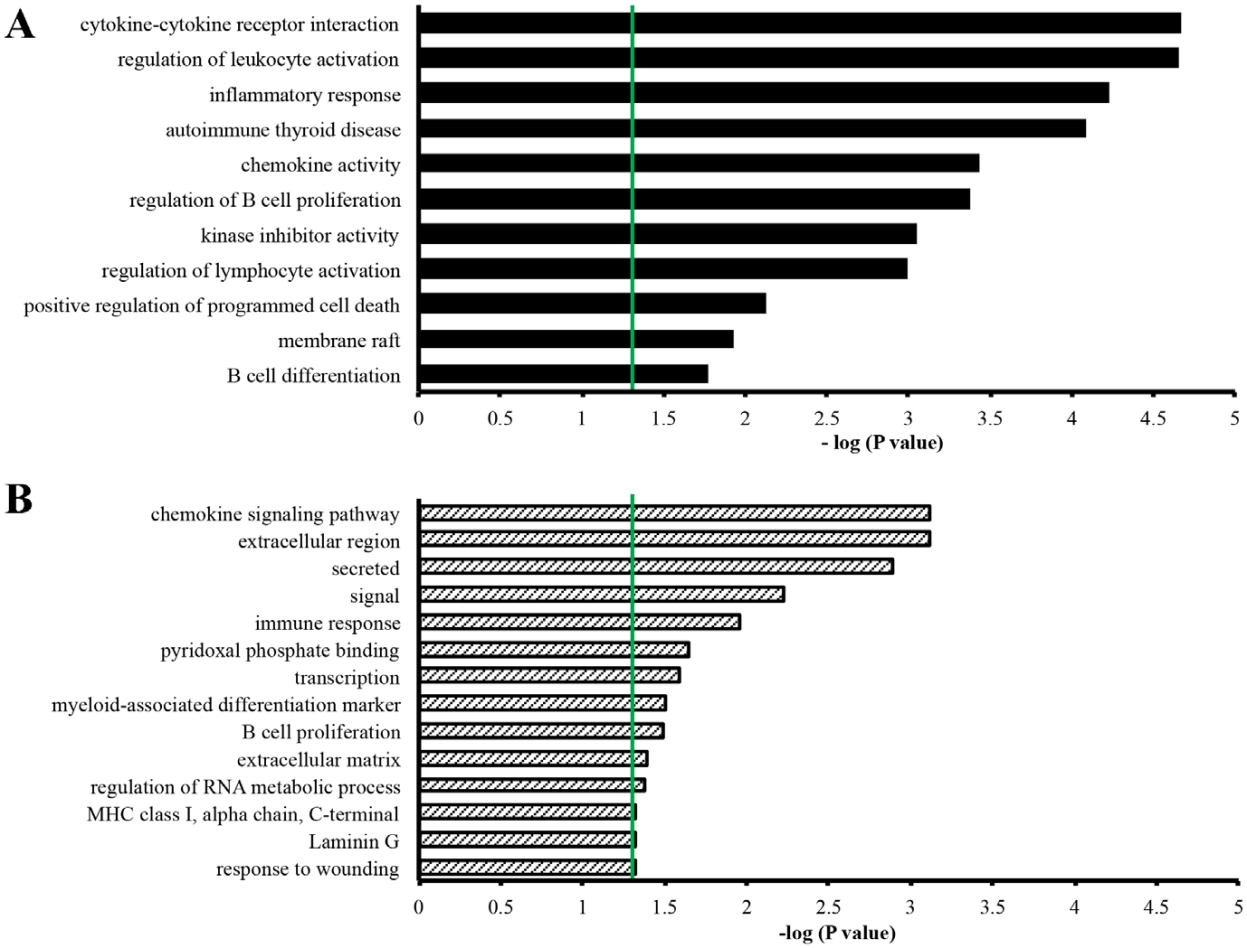
Gene enrichment analysis of DEGs specific of the clinical and preclinical stage of the disease. The most relevant GO terms (y axis) associated to clinical (**A**) and preclinical (**B**) phase are listed according to decreasing statistical significance from top to bottom. The threshold for statistical significance is marked by the green lines. The enrichment analysis was performed using DAVID bioinformatics tool 6.7 (NIAID/NIH, USA).

A gene enrichment analysis was performed to identify the most enriched GO terms in the clinical and preclinical groups (Fig 2). DEGs specific of the clinical group were clustered in functional categories related to cytokine-cytokine receptor interaction, regulation of leukocyte activation, inflammatory response, autoimmune thyroid disease, chemokine activity, B cell proliferation and differentiation, regulation of apoptosis, kinase inhibitor activity, and membrane raft (Fig 2B). The preclinical stage was characterized by enrichment in gene clusters related to chemokine signaling pathway, extracellular region, secreted protein, immune response, pyridoxal phosphate binding, transcription, myeloid-associated differentiation marker, B cell proliferation, extracellular matrix, RNA metabolic process, MHC class I, Laminin G and response to wounding (Fig 2A).

**Fig 3:**
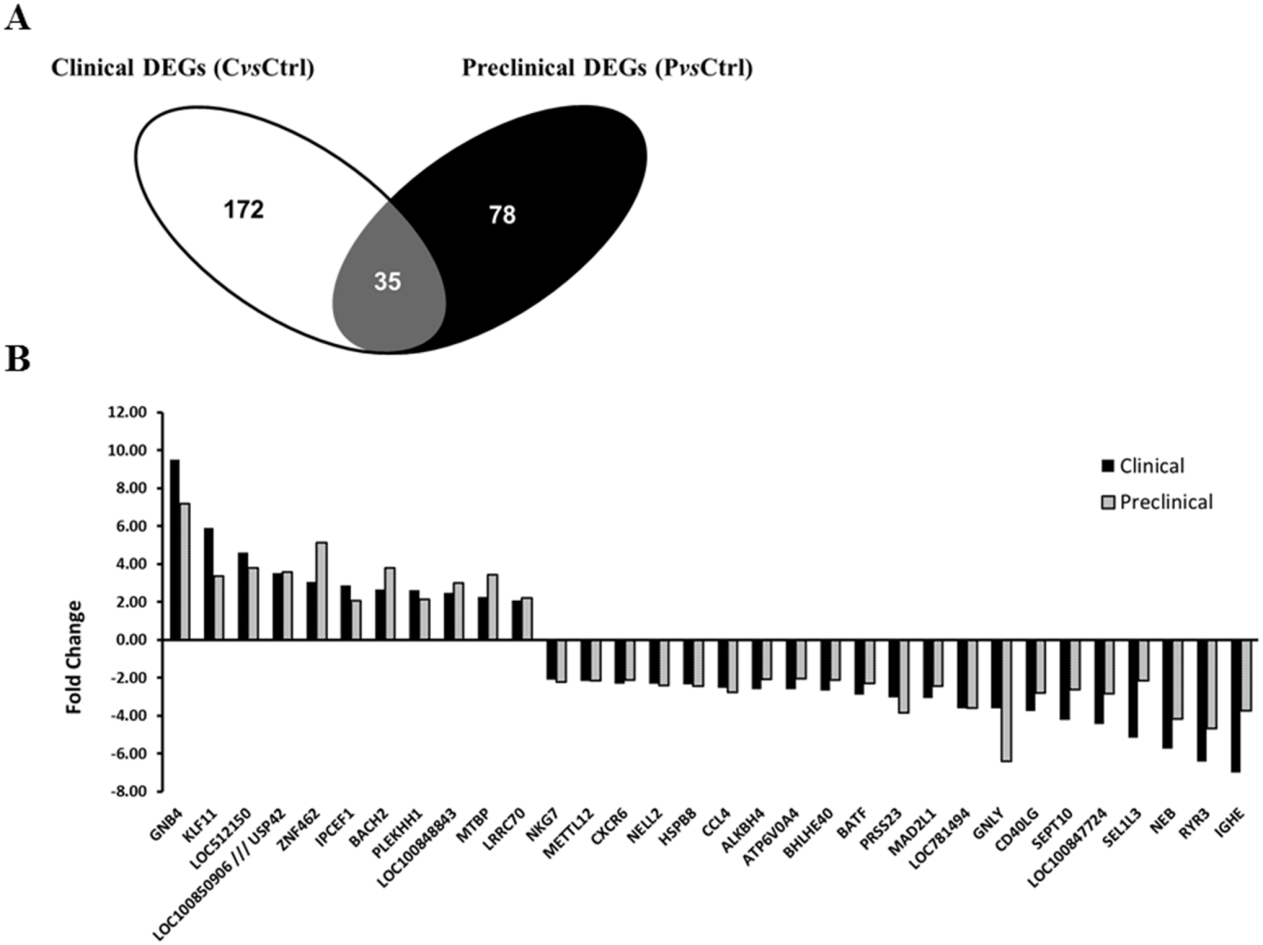
Identification of common DEGs in blood of preclinical and clinical atypical BSE-infected cattle. (A) Venn diagram showing the number of differentially expressed probe sets in blood of clinical and preclinical cattle. The intersection in grey represents 35 differentially expressed probe sets corresponding to 32 differentially expressed genes (DEGs) that are in common between the two stages of the disease. (B) Expression pattern of the common 32 DEGs. The histograms represent the fold change relative to the control group. PvsCtrl = preclinical versus control, CvsCtrl = clinical versus control.

When comparing the differentially regulated probe sets identified in the preclinical and clinical groups, it was found that 35 differentially expressed probe sets (corresponding to 32 DEGs) were common between the two stages of disease (Fig 3), leaving 172 DEGs specific to clinical and 78 genes specific to preclinical animals. Remarkably, all of the 32 common DEGs displayed a very similar pattern of expression in the clinical and preclinical groups, as shown in Fig 3B. These genes are listed in bold in S1 and S2 Tables.

To further dissect gene expression alterations during the progression of the disease, we performed a statistical analysis to identify specific changes between the clinical and preclinical stages (CvsP). Indeed, we found 235 DEGs, which were significantly enriched in pathways related to immune response (regulation of B cell proliferation, leucocyte activation, ISG15-protein conjugation and chemokine signaling were among the most significant). The list of the most relevant enriched probe sets can be found in S3 Table.

**Fig 4:**
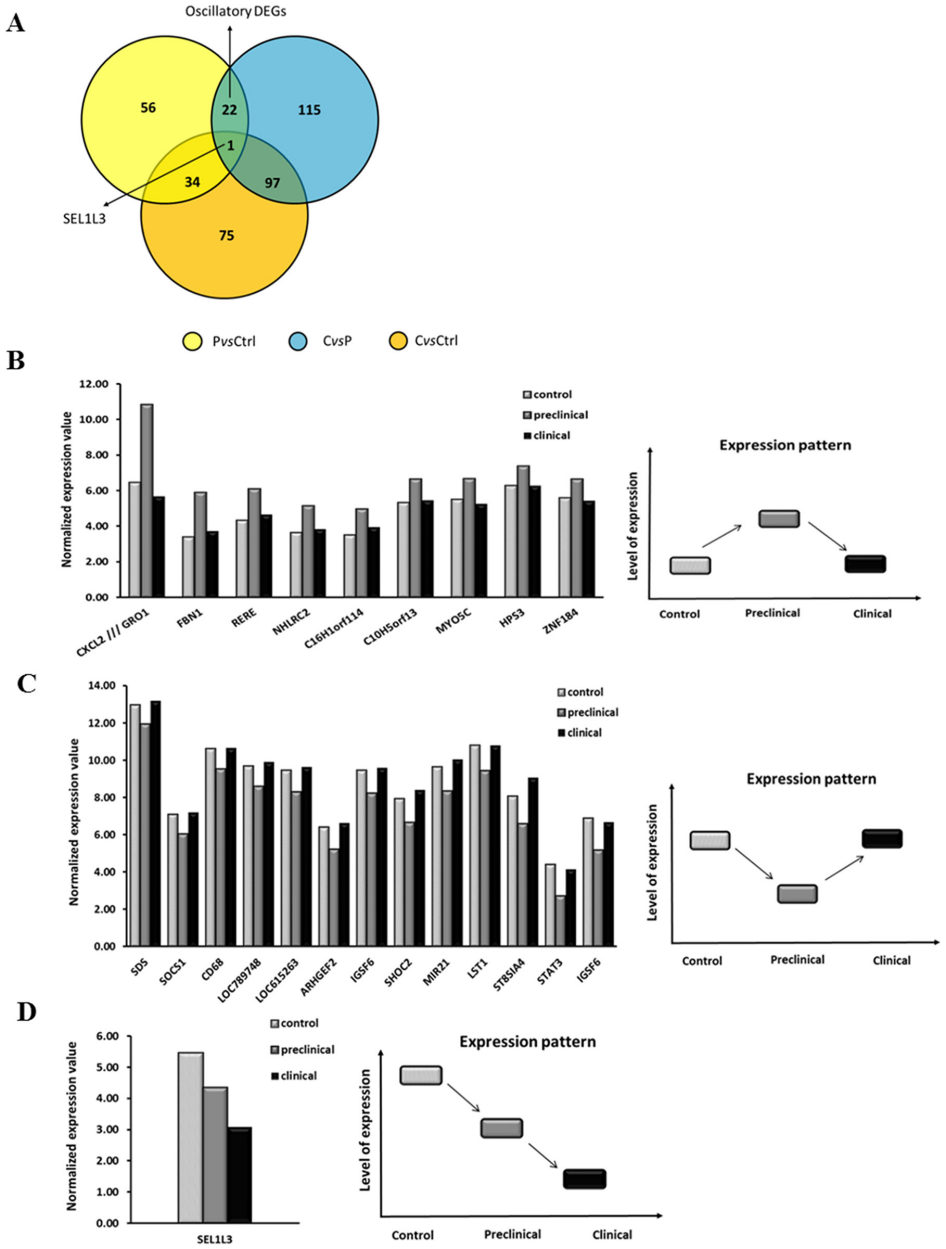
DEGs in common between CvsP, PvsCtrl and CvsCtrl comparisons. (A) Venn diagram revealed the presence of 22 DEGs in common between CvsP and PvsCtrl comparisons. One DEG (SEL1L3, Sel-1 Suppressor Of Lin-12-Like 3) was common in the three comparisons. (B-C) The normalized expression values of the 22 common DEGs for CvsP and PvsCtrl comparisons are represented by the histograms. As indicated by the schematic figures on the right, these 22 DEGs showed an oscillatory pattern of expression: (B) 9/22 were upregulated in PvsCtrl and then downregulated in CvsP comparisons, while (C) 13/22 were downregulated in PvsCtrl and upregulated in CvsP comparison, respectively. (D) SEL1L3, the only gene found in common among the three comparisons (CvsP, CvsCtrl and PvsCtrl), showed a progressive downregulation during the preclinical and the clinical phase of the infection. P = preclinical, C = clinical and Ctrl = control, *vs* = *versus*. DEGs = differentially expressed genes.

We used a Venn diagram to compare the DEGs found in PvsCtrl, CvsCtrl and CvsP analyses that were previously performed and then we examined the expression levels of common DEGs (Fig 4). Venn diagram revealed the presence of one DEG in common between PvsCtrl, CvsCtrl and CvsP comparisons, while 22 genes were differentially expressed in PvsCtrl and CvsP but not in CvsCtrl comparisons (Fig 4A). We found that these 22 DEGs had an opposite fold change sign in PvsCtrl and CvsP, thus indicating an oscillatory pattern of expression (see Fig 4B, 4C and Table 3). In particular, 9 out of 22 DEGs were up-regulated in the preclinical phase and then went back roughly to the expression level of the controls in the clinical stage (Fig 4B). The remaining 13 out of 22 DEGs were down-regulated in the preclinical phase and then went back almost to control levels in the clinical phase (Fig 4C). The only gene in common between the three comparisons, namely Sel-1 Suppressor Of Lin-12-Like 3 **(***SEL1L3*), was progressively down-regulated during the preclinical and the clinical phase of the infection, as shown in Fig 4D.

**Table 3:** List of DEGs characterized by an up-down/down-up pattern of expression.^a^

Probe Name	Gene Symbol	Gene Name	Fold Change in PvsCtrl	Fold Change in CvsP
Bt.611.1.S2_at	CXCL2	chemokine (C-X-C motif) ligand 2	20.96797	-35.4276
Bt.5021.1.S1_at	FBN1	fibrillin 1	5.660874	-4.47422
Bt.18013.1.A1_at	RERE	arginine-glutamic acid dipeptide (RE) repeats	3.399468	-2.66838
Bt.18234.1.S1_at	IGSF6	immunoglobulin superfamily, member 6	-3.28943	2.811553
Bt.15334.2.A1_at	STAT3	Signal transducer and activator of transcription 3	-3.23829	2.678539
Bt.20833.3.A1_at	NHLRC2	NHL repeat containing 2	2.825469	-2.43499
Bt.27161.1.A1_at	C16H1orf114	chromosome 16 open reading frame, human C1orf114	2.782753	-2.03538
Bt.17789.1.A1_at	ST8SIA4	ST8 alpha-N-acetyl-neuraminide alpha-2,8-sialyltransferase 4	-2.77122	5.521504
Bt.21221.1.S1_at	LST1	leukocyte specific transcript 1	-2.55548	2.531317
Bt.3780.1.S1_at	C10H5orf13	chromosome 10 open reading frame, human C5orf13	2.480665	-2.2476
Bt.9841.1.S1_at	MIR21	microRNA mir-21	-2.45176	3.249144
Bt.18474.2.A1_at	SHOC2	soc-2 suppressor of clear homolog (C. elegans)	-2.4061	3.30272
Bt.21868.2.S1_at	IGSF6	immunoglobulin superfamily, member 6	-2.34146	2.567908
Bt.27421.1.S1_at	ARHGEF2	Rho/Rac guanine nucleotide exchange factor (GEF) 2	-2.26254	2.6399
Bt.16088.1.A1_at	MYO5C	myosin VC	2.260998	-2.64645
Bt.1711.1.A1_at	LOC615263	uncharacterized LOC615263	-2.20341	2.511818
Bt.22390.1.S1_at	HPS3	Hermansky-Pudlak syndrome 3	2.146785	-2.13082
Bt.1847.1.S1_at	LOC789748	Sialic acid-binding Ig-like lectin 14-like	-2.12509	2.458681
Bt.2334.1.S1_at	CD68	CD68 molecule	-2.11618	2.156622
Bt.26430.1.S1_at	ZNF184	zinc finger protein 184	2.099151	-2.31926
Bt.1736.1.A1_at	SOCS1	suppressor of cytokine signaling 1	-2.06053	2.221149
Bt.5878.2.S1_at	SDS	serine dehydratase	-2.02673	2.367767

^a^ For each gene the fold changes found in PvsCtrl (preclinical versus control) and CvsP (clinical versus preclinical) comparisons are reported.

### Validation of Microarray Results by RT-qPCR

To confirm the microarray results, RT-qPCR analysis was performed using the SYBR^®^ green assay. A normalization factor based on three reference genes (glyceraldehyde-3-phosphate dehydrogenase, *GAPDH*; ribosomal protein L12, *RPL12*; actin, beta, *ACTB*) was used for the analysis. The stability of the selected reference genes was determined by calculating their geNorm M value (M) and the coefficient of variation (CV) on the normalized relative quantities (S2 Appendix file) [50]. 9 DEGs related to different functional categories were chosen for the validation: *GNLY* (granulysin), *CD40L* (CD40 ligand), *PDK4* (pyruvate dehydrogenase lipoamide kinase isozyme 4), *IDO1* (indoleamine 2, 3-dioxygenase 1), *HBA2* (hemoglobin, alpha 2), *XIST* (X-inactive specific transcript), *GNB4* (guanine nucleotide binding protein beta polypeptide 4), *BOLA* (MHC class I heavy chain), and *SEL1L3* (Table 4).

**Table 4:** Genes analyzed by RT-qPCR^a^.

Gene	Primer sequence		Amplicon length (bp)	Accession number
ACTB*	F:	GGA CTT CGA GCA GGA GAT GG	148	NM_173979.3
	R:	TTC CAT GCC CAG GAA GGA AG		
GAPDH*	F:	AGG TCG GAG TGA ACG GAT TC	85	NM_001034034.2
	R:	ATG GCG ACG ATG TCC ACT TT		
RPL12*	F:	AGG GTC TGA GGA TTA CAG TGA AA	83	NM_205797.1
	R:	GAT CAG GGC AGA AGC AGA AGG		
CD40L	F:	ACA ACC TCT GTT CTC CAG TG	82	NM_174624.2
	R:	GTC GTT TCC CGT TTT CGA GG		
XIST	F:	GTG GCA AGG ACC AGA ATG GA	112	NR_001464.2
	R:	TCC GAC CCC AGT ATT AGC CA		
GNLY	F:	AGC CCG ATG AGA ATA CCG TT	120	NM_001075143.1
	R:	CGA TGT CCT CAG CGA TGG TA		
PDK4	F:	TGG TGT TCC CCT GAG AGT CA	109	NM_001101883.1
	R:	GTA ACC AAA ACC AGC CAG CG		
HBA2	F:	ACA AGG GCA ATG TCA AGG CC	124	NM_001077422.3
	R:	TCG AAG TGG GGG AAG TAG GT		
GNB4	F:	AGA TCG TGC AGG TGT TCT TG	96	NM_001099033.1
	R:	CTG TCC CAA GAC CCT GTT G		
IDO1	F:	ATT GGT GGA GTC CCT GCA GAC	150	NM_001101866.2
	R:	CTG CAG GGT AGC ATT GCC TT		
BOLA	F:	CTC GTA GTA GCT GTG GTG GC	96	NM_001038518.1
	R:	ACT GTC ACT GCT TGC AGC C		
SEL1L3	F:	TGA AGG AGT GGTTTC GCC TG	79	NM_001206556.2
	R:	TTC AAA TCC TGC CCA GTG CT		

^a^ Primers (F, forward; R,reverse) used for gene amplification, amplicon size, and GenBank^®^ accession numbers for the bovine cDNA sequences used for primer design. All primers were designed according to the genome sequence of *Bos taurus*.

**Fig 5:**
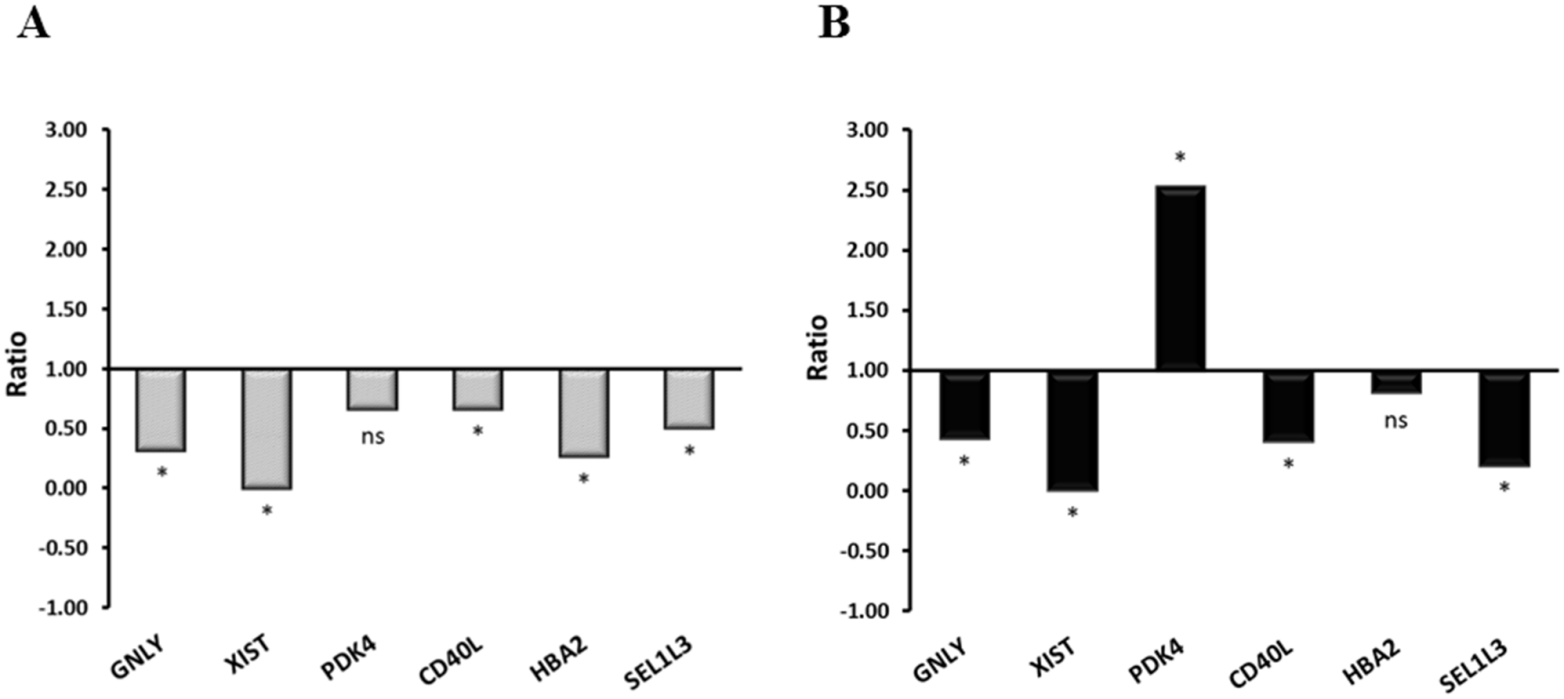
Validation of microarray data by RT-qPCR. Differential expression of selected genes in blood from preclinical (A) and clinical (B) atypical BSE-infected cattle. Ganulysin (GNLY), X-inactive specific transcript (XIST), pyruvate dehydrogenase kinase 4 (PDK4), CD40 ligand (CD40L), haemoglobin, alpha 2 (HBA2) and Sel-1 Suppressor Of Lin-12-Like 3 protein (SEL1L3). Gene expression (ratio) values are represented as relative to RNA levels in control animals. Ns = not significant; *P value ≤ 0.05.

These genes were selected on the basis of their fold changes, *p* values and relevance in the literature. Moreover, because we performed several statistical analyses of the microarray data, we chose genes that appeared as differentially expressed with the highest frequency in different resulting datasets. Despite microarray *p value* for *HBA2* was not significant, this gene was selected for the RT-qPCR validation since a previous work published by our group highlighted its involvement in prion pathogenesis [51]. The RT-qPCR analysis confirmed the microarray results for six out of nine genes selected (*XIST*, *CD40L*, *GNLY*, *PDK4*, *HBA2* and *SEL1L3*), which are represented in Table 5 and Fig 5: *CD40L*, *XIST*, *SEL1L3* and *GNLY* downregulation was confirmed in both preclinical and clinical groups, while *HBA2* was significantly down-regulated only in preclinical animals. Significant *PDK4* upregulation was found in the clinical stage, but not in the preclinical one. These results were in line with the microarray data (Table 5).

**Table 5:** Differential expression of selected genes quantified by microarray and RT-qPCR analysis ^a^.

Gene name	Gene symbol	Microarray fold change		RT-qPCR ratio/FC	
		preclinical	clinical	preclinical	clinical
Granulysin	GNLY	-6.318	-3.698	0.322/-3.106	0.433/-2.309
X-inactive specific transcript	XIST	-33.240	-22.612	0.003/-333.333	0.004/-250.000
Pyruvate dehydrogenase kinase 4	PDK4	1.752 (ns)	5.793	0.665/ -1.503 (ns)	2.536
CD40 ligand	CD40L	-2.786	-3.761	0.667/-1.499	0.408/-2.450
Haemoglobin, alpha 2	HBA2	-3.866 (ns)	-2.259 (ns)	0.269/-3.717	0.816/-1.225 (ns)
Suppressor Of Lin-12-Like Protein 3	SEL1L3	-2.16	-5.17	0.504/-1.984	0.213/-4.695

^a^ For an easier interpretation, the differential expression of the downregulated genes measured by RT-qPCR is reported both as original ratio and as fold change calculated as -1/ratio. Ns = not significant

## Discussion

Whole blood is the most suitable tissue for a prospective rapid diagnostic test since it minimizes sample handling artifacts and reduces sample variability due to fractionation. The present study revealed a substantial gene expression alteration in whole blood from atypical BSE-infected cattle, which could be investigated in future experiments and, if confirmed, could be exploited as a signature for the disease. One of the major caveats in using peripheral blood is that its cellular components may change dramatically during infections or inflammation. The animals used in the present study did not show any apparent side pathology, they were monitored daily by the husbandry staff and their blood was examined for serum aspartate aminotransferase (AST), creatine kinase (CK) and manganese [23]. Nonetheless, possible interference due to hidden pathologies or to inter-individual variations in hematocrit and white blood cell count may affect interpretation of expression data and should be considered as an important variable in future studies. In light of this, the present results should be read as a first exploration of whole blood transcriptomics during a prion infection. To our knowledge, this is the first microarray study of whole blood from BSE-infected cattle. Indeed, in a study published by Panelli *et al*. [36], fractionated white blood cells were analyzed to detect gene expression changes in L-type infected animals. Very few DEGs are common between the two studies. However, this discrepancy may be explained by different infection methods, microarray platforms, statistical analysis stringency and *p* value cut-off. Also, the white cells used in the study of Panelli *et al*. were isolated from 1 year post-infection animals, while in our study we used whole blood from preclinical and clinical infected cattle (around 6 months and 22–26 post-infection respectively).

In the present study, 4 statistical comparisons were performed: infected (preclinical and clinical) versus control (IvsCtrl), preclinical versus control (PvsCtrl), clinical versus control (CvsCtrl) and clinical versus preclinical (CvsP) comparisons. Since our goal was to find a common pattern among all the atypical BSE-infected cattle, we defined them as a single group of infected (H-type and L-type) animals. Indeed, as published by Konold and colleagues [23], these animals shared a very similar phenotype in terms of behavioral and clinical signs. Also, another study by Priemer and colleagues indicated a large similarity at level of PrP^Sc^ anatomical distribution for the atypical strains, with only slight differences in the overall intensities between H- and L-type [27]. Nonetheless, even if H- and L-type BSE are reported to share many similarities, they constitute two distinct BSE variants which are characterized by a different electrophoretic mobility of PrP^Sc^ unglycosylated moiety after proteinase K (PK) digestion [16, 18]. Statistical comparison between the 4 H-type and the 4 L-type infected animals was carried out in preliminary analyses (see S4 Table), but only a limited number of DEGs was found. Among them, only 15 had a p value lower than 0.01 and only 16 showed a fold change higher than 3, indicating that, at least in terms of number of DEGs, these two groups did not display large differences. For these reasons, we decided to focus our attention on finding a common gene pattern among all the atypical BSE-infected cattle and therefore we pooled the two groups. Due to the high inter-animal variability, which is expected for outbreed animals, further studies in larger animal cohorts would be required to investigate in detail the strain-specific gene expression changes occurring during the progression of the disease. Still, the HvsL analysis can be used as a sort of internal control in this study.

Another aspect to be taken into account when reading the present results is that additional negative control cattle, aged from 12 to 37 months and derived from a different herd compared to the Konold’s study groups [23], were introduced in the analyses. The addition of these controls was useful to balance the samples from infected animals and allowed a preliminary exploration of the differentially expressed transcripts. However, age-related and environmental variability may have affected in some degree the data and need to be considered for their correct interpretation. Despite some limitations, since several statistical analyses were performed (including the CvsP analysis, in which all the animals derived from the same herd) a cross comparison of all them, as we did with the Venn Diagram, may be very useful in order to define a set of genes which could be a good starting point for further validation experiments in the future. In the first statistical analysis we performed (IvsC), we found that among 101 DEGs, 93 had known functions and were involved in several biological processes and molecular pathways, such as autoimmune thyroiditis, chemokine and cytokine activity, regulation of the secretion pathway, the immune system and antigen presentation[52]. Previous studies on CNS tissues from BSE-infected animals also showed the involvement of many of these pathways in prion pathogenesis [4, 35, 52]. This similarity between brain and blood may not be surprising, since it has been shown in the literature that blood transcriptome analyses identify genes that are relevant to the pathological processes occurring in the CNS [53]. Indeed, measuring disease-related gene expression in peripheral blood may be a useful proxy measure for gene expression in the CNS [53, 54].

To characterize the gene expression profile in the preclinical and clinical stages, we performed the PvsCtrl and the CvsCtrl statistical comparisons. We found that 113 probe sets were differentially regulated in the preclinical stage of the disease, while 207 probe sets had an altered expression in the clinical phase. Importantly, the present results indicated that, at least in blood, a consistent gene expression alteration is present from the early stages of the disease. This finding is in agreement with microarray analysis carried out by Tang *et al*., which revealed the highest degree of differential gene regulation in brains of cBSE-infected cattle at 21 months post infection, which is prior to the detection of infectivity [4]. Also, Tortosa and colleagues found a significant number of DEGs at early stages of the disease in the CNS from cBSE-infected transgenic mice [52].

Venn diagram analysis revealed that 32 DEGs were in common between the clinical and preclinical groups and, remarkably, they had a very similar pattern of expression in both stages of the disease. Since these genes are altered in both phases, it would be very interesting to confirm their differential expression in future experiments with additional negative controls, and eventually in blood from human patients.

Based on GO enrichment analysis, we found that immunity and inflammation processes were strongly involved during the progression of the disease stages. Interestingly, we found that antigen processing and presentation via MHC (major histocompatibility complex) molecules and the autoimmune thyroiditis pathway were significantly altered in atypical BSE-challenged animals. The majority of MHC class I molecule coding-genes were down-regulated in infected cattle (three out of four probes) and, also, MHC class II molecule coding transcripts were found to be down-regulated during the progression of the clinical signs (four out of four probes were down-regulated in the CvsP comparison). The involvement of MHC transcripts in prion pathogenesis is supported by another microarray study published by Khaniya and colleagues in 2009 [55]. In line with the trend found by the microarray analysis, the RT-qPCR validation experiments indicated a downregulation for MHC class I heavy chain (*BOLA*), even though the results failed to reach the statistical significance (data not shown).

Regarding the autoimmune thyroiditis pathway, it is well known in the literature that Hashimoto’s encephalitis, together with the associated thyroiditis, is a differential diagnosis for CJD, since the two pathologies share a very similar clinical symptomatology [56]. As hypothesized previously by Prusiner and colleagues, the clinical and neuropathological similarities between CJD and Hashimoto’s thyroiditis raise the possibility that protein misprocessing may underlie both neurodegenerative and autoimmune diseases [57].

Finally, a fourth statistical analysis was performed to identify any specific changes between the clinical and the preclinical stages of disease (CvsP). Indeed, we found that the last phases of the disease are accompanied by the overactivation of several genes involved in the immune defense response. In particular, the shift from the preclinical towards the clinical stage was characterized by the upregulation of genes involved in B cell proliferation and the ISG15 (IFN-induced 15-kDa protein) conjugation system. ISG15 is a ubiquitin-like molecule that is tightly regulated by specific innate immunity signaling pathways [58]. Interestingly, it has been shown in the literature that this protein is over-activated in the spinal cord of amyotrophic lateral sclerosis mice models [59] and it has been indicated as a general marker for both acute and chronic neuronal injuries [60].

To further analyze the data, we compared the list of DEGs found in PvsCtrl, CvsP and CvsCtrl and found 22 genes with an oscillatory pattern of expression, being differentially expressed in the preclinical stage and then going back roughly to the control level in the clinical stage. Interestingly, some of the oscillatory DEGs are involved in regulation of transcription, thus suggesting that the gene expression during atypical BSE infection is tightly regulated. Venn diagram analysis revealed that one gene, *SEL1L3*, was down-regulated in all the comparisons (PvsCtrl, CvsCtrl, CvsP). SEL1L3 codes for a transmembrane protein whose function is unknown. Interestingly, an important paralog of *SEL1L3*, *SEL1L*, is involved in the retrotranslocation of misfolded proteins from the lumen of the endoplasmic reticulum to the cytosol, where they are degraded by the proteasome in an ubiquitin-dependent manner [61]. Therefore, we could hypothesize that its down-regulation in prion infected animals would lead to a reduced degradation of PrP^Sc^, thus supporting the progression of the disease. We validated this gene by RT-qPCR, confirming its downregulation in both the preclinical and clinical stages of the disease. Further investigation on the function of *SEL1L3L* would be of great interest since this gene may play an important role in prion disease and maybe other neurodegenerative illnesses.

Besides *SEL1L3*, five other genes were validated by RT-qPCR; here we will briefly discuss how these genes may be involved in prion pathogenesis and in host response to prion infection.

*GNLY* and *CD40L* were found to be down-regulated in both preclinical and clinical stages. GNLY is a powerful antimicrobial protein contained within the granules of cytotoxic T lymphocyte and natural killer cells. This gene was found to be downregulated also in a microarray study performed on the medulla oblongata from sheep with preclinical natural scrapie [62]. Thus, it may be a good candidate as an early biomarker for atypical BSE but also for other prion diseases.

CD40–CD40L interactions mediate a broad variety of immune and inflammatory responses and have been implicated in the pathogenesis of Alzheimer’s disease (AD) [63, 64]. Although the importance of *CD40L* in prion disease progression has not yet been clarified (66–68), its downregulation in blood during both preclinical and clinical stages of atypical BSE-infection suggests that prion infection has an impact on the host immune system response and that immune tolerance may be an active process induced by prions.

Two other downregulated genes were validated by RT-qPCR, namely *HB*A2 and *XIST*. Concerning *HBA2*, we found a downregulation in preclinical atypical BSE-infected cattle. Haemoglobins are iron-containing proteins that transport oxygen in the blood of most vertebrates. Beside blood, *HBA* and *HBB* are also expressed in mesencephalic dopaminergic neurons and glial cells [65] and are down regulated in AD, PD and other neurodegenerative diseases [66]. Haemoglobin genes expression alteration during preclinical scrapie was also found in the spleen and CNS of infected animals [67, 68], as well as in the brains of nonhuman primates infected with BSE [51]. These findings suggest an involvement of these genes in the host response to general neurodegenerative processes. Besides changes in transcript levels, it has been found that both HbA and HbB protein distribution is altered in mitochondrial fractions from PD degenerating brain [69]. Moreover, HbA is also expressed in endothelial cells, where it regulates the nitric oxide signaling [70]. Even though a clear mechanism linking these molecules to neurodegeneration has not yet been described, taken together these findings strongly suggest a central role for haemoglobin in neurodegenerative processes.

A marked downregulation in *XIST* expression was found in our infected animals. *XIST* is a gene located on X chromosomes which codes for a long non-coding RNA (LncRNA) involved in X-chromosome dosage control [71, 72]. LncRNAs are emerging as useful biomarkers for neurodegenerative diseases such as AD [73] and other disease processes [74], and they can be easily detected in blood and urine from patients. In addition, we cannot exclude the possibility that the alteration in *XIST* expression may have some role in gender-dependent response to prion infection [75].

RT-qPCR experiments confirmed the upregulation of *PDK4* in clinically affected animals. *PDK4* encodes for a mitochondrial protein involved in glucose metabolism through the inhibition of pyruvate dehydrogenase complex, which leads to a reduction in pyruvate conversion to acetyl-CoA [76]. In the literature, a key role has been suggested for acetyl-CoA fueling for the survival of cholinergic neurons in the course of neurodegenerative diseases [77]. *PDK4* overactivation can lead to a switch from glucose catabolism to fatty acid utilization [78], thus increasing the production of ketone bodies. Notably, it has been shown in the literature that these molecules are able to cross the blood brain barrier. We could speculate that in prion infection (or at least in atypical BSE infection) the concentration of ketone bodies would rise in blood, as a consequence of PDK4 upregulation, and act in the brain as neuroprotective molecules [79, 80]. This would be an attempt by the organism to prevent the neurodegeneration induced by prions.

## Conclusions

In conclusion, the present study has led to the identification of several gene expression changes in whole blood from clinical and preclinical atypical BSE cattle, which upon further investigation and validation in blood from human patients, might represent a molecular fingerprint to characterize this disease. By comparing our results with other studies on various animal prion diseases, we observed that some of the most significantly altered DEGs we found in blood were found differentially expressed also in brain tissue from BSE-infected cattle; this observation indicates that whole blood transcriptome analyses may serve as a proxy measure for the changes occurring in the CNS of infected animals. Furthermore, our study underlines the importance of utilizing whole blood, without any additional manipulation, as a source tissue as it is an easily accessible body fluid. In addition, the transcription regulation activated in atypical BSE infections is similar to some extent to the one observed in the literature for cBSE, even though the clinical characteristics and biochemical properties are very different. Thus, this gene expression profile may be investigated in other BSE infections to identify a common molecular fingerprint.

Overall, our study confirmed the differential expression of 6 genes (*XIST*, *CD40L*, *GNLY*, *PDK4*, *HBA2* and *SEL1L3*), which may play several roles in atypical BSE pathogenesis and, possibly, in other prion infections. Indeed, they are involved in multiple pathways such as immune response, inflammation, and glucose catabolism. Even though further studies are required to investigate the specific involvement of all the identified genes in prion diseases, our data indicate an important role for immune system regulation in the prion pathogenesis of atypical BSE and maybe in BSE as well as in other prion diseases in general.

## Supporting Information

S1 Appendix Post hybridization quality assessment. (**A**) Normalized unscaled standard error (NUSE), (**B**) relative log expression (RLE) and (**C**) raw signal intensity plots are used to check for technical problems and to spot outlier samples after GCRMA normalization. Box plots centered higher than normal (typically above 1.1 in the NUSE plot) and/or having a larger spread in the RLE plots represent arrays with quality problems. One outlier was easily identified by post hybridization quality assessment (black arrow in panel A and B, control sample cS5).
(TIF)

S2 Appendix Reference gene expression stability. Stability of the selected reference genes was determined by calculating their geNorm M value (M) (**A**) and the coefficient of variation (CV) (**B**) on the normalized relative quantities (CNRQ). The dashed green lines in panel A and B indicate the maximum acceptable threshold for M and CV values, respectively. These thresholds have been empirically determined by previous experiments performed by Hellemans et al. (see ref. 42).
(TIF)

S1 Table Differentially expressed genes found in preclinical animals versus controls (PvsCtrl) by the microarray analysis. FC = fold change. Common DEGs between preclinical and clinical animals are highlighted in bold.
(XLSX)

S2 Table Differentially expressed genes found in clinical animals versus controls (CvsCtrl) by the microarray analysis. FC = fold change. Common DEGs between preclinical and clinical animals are highlighted in bold.
(XLSX)

S3 Table Functional classification of differentially expressed genes found in blood of clinical versus preclinical animals. The gene enrichment analysis was performed using DAVID bioinformatics tool 6.7 (NIAID/NIH, USA). Only genes with a known GO are represented in the list. The DEGs which fell in more than one category for simplicity are presented under a single functional heading. FC and P values refer to the microarray analysis.
(XLSX)

S4 Table List of DEGs found in H*vs*L statistical comparison. FC = fold change.
(XLSX)
