# Supplementary figures and images for: Whole Blood Gene Expression Profiling in Preclinical and Clinical Cattle Infected with Atypical Bovine Spongiform Encephalopathy
Source: PLoS One. 2016 Apr 13;11(4):e0153425. doi: 10.1371/journal.pone.0153425 (PMC4830546; doi:10.1371/journal.pone.0153425)

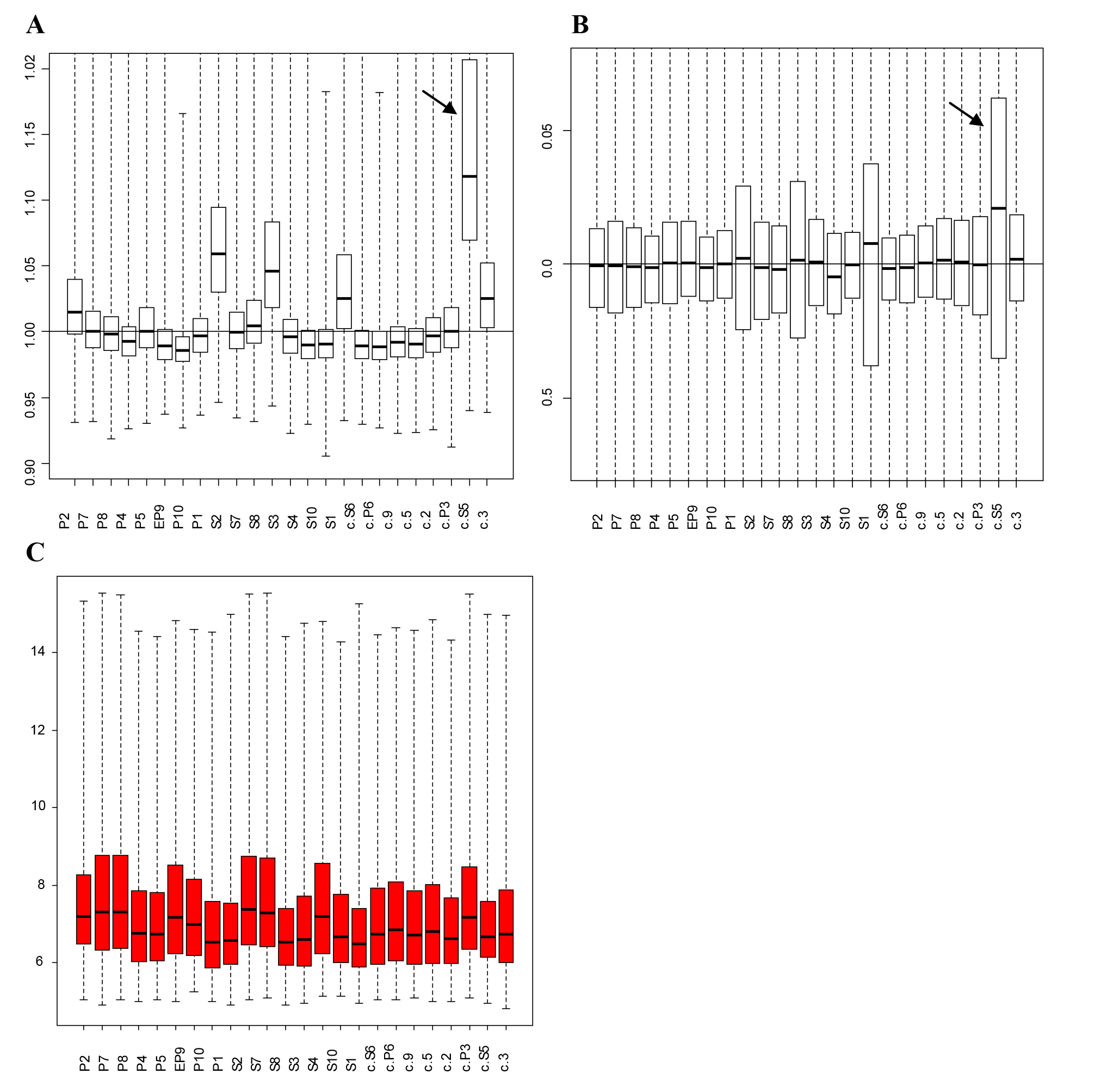

Supplement: S1 Appendix — (A) Normalized unscaled standard error (NUSE), (B) relative log expression (RLE) and (C) raw signal intensity plots are used to check for technical problems and to spot outlier samples after GCRMA normalization. Box plots centered higher than normal (typically above 1.1 in the NUSE plot) and/or having a larger spread in the RLE plots represent arrays with quality problems. One outlier was easily identified by post hybridization quality assessment (black arrow in panel A and B, control sample cS5). (TIF) [file pone.0153425.s001.tif]

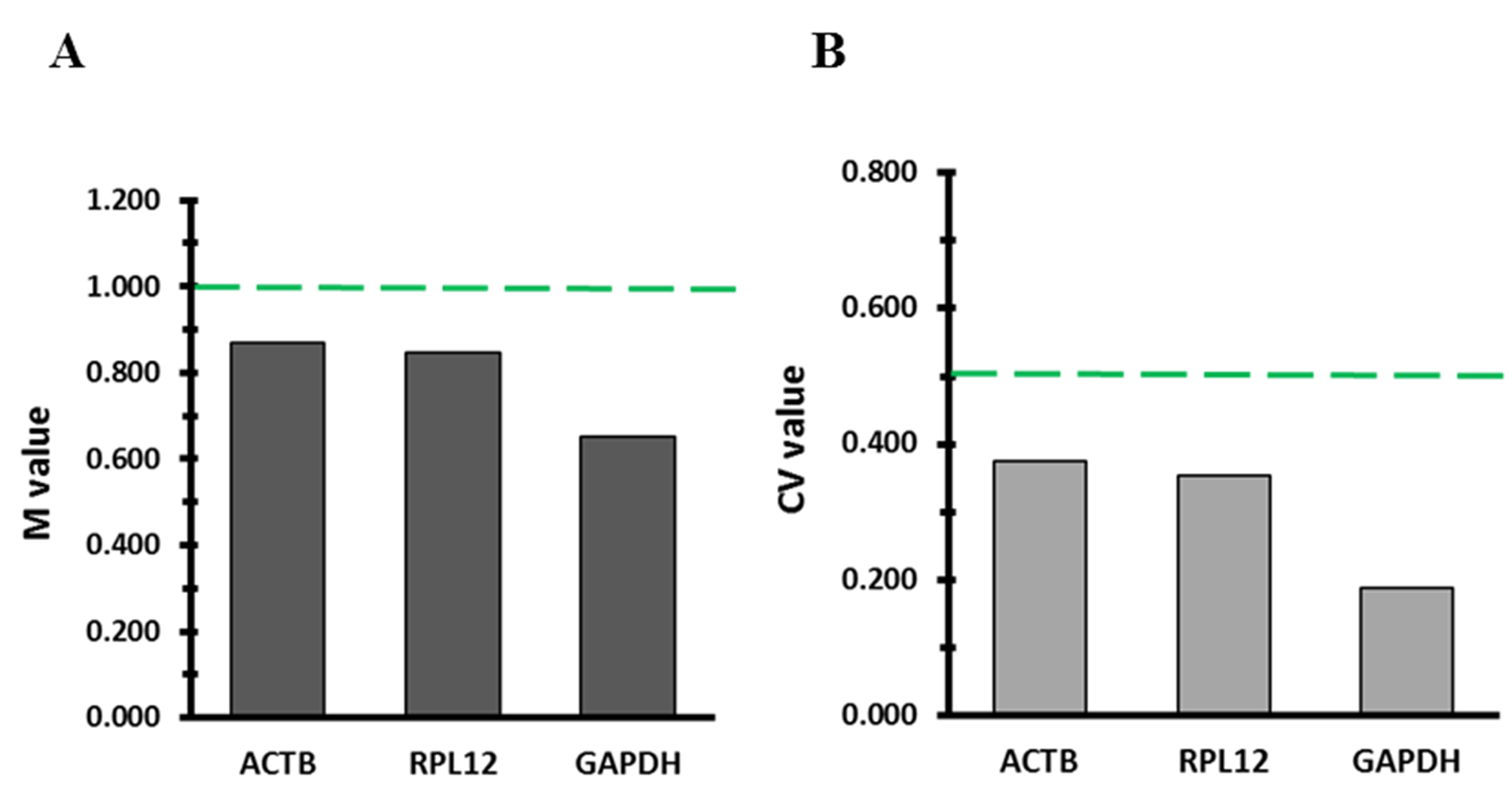

Supplement: S2 Appendix — Stability of the selected reference genes was determined by calculating their geNorm M value (M) (A) and the coefficient of variation (CV) (B) on the normalized relative quantities (CNRQ). The dashed green lines in panel A and B indicate the maximum acceptable threshold for M and CV values, respectively. These thresholds have been empirically determined by previous experiments performed by Hellemans et al. (see ref. 42). (TIF) [file pone.0153425.s002.tif]
